# Is vaccination a feasible public health strategy against fatal Borna disease virus 1 (BoDV-1) encephalitis? An epidemiological perspective

**DOI:** 10.1371/journal.ppat.1013571

**Published:** 2025-10-13

**Authors:** Kirsten Pörtner, Christina Frank, Hendrik Wilking, Klaus Stark, Christiane Herden, Martin Beer, Dennis Rubbenstroth, Dennis Tappe

**Affiliations:** 1 Infectious Disease Epidemiology, Robert Koch Institute, Berlin, Germany; 2 Institute for Veterinary Pathology, Justus Liebig-University Gießen, Gießen, Germany; 3 Institute of Diagnostic Virology, Friedrich Loeffler Institute, Greifswald- Insel Riems, Germany; 4 Reference Laboratory for Bornaviruses, Bernhard Nocht Institute for Tropical Medicine, Hamburg, Germany; University of Wisconsin-Madison, UNITED STATES OF AMERICA

## Abstract

Human Borna disease virus 1 (BoDV-1) encephalitis is characterized by rapid clinical progression, an absence of a causal therapy and an extremely high case fatality rate. Here, we discuss prevention options through a hypothetical vaccine focusing on epidemiological features.

## Zoonotic Borna disease virus 1 (BoDV-1) causes fatal human encephalitis

**Fig 1:**
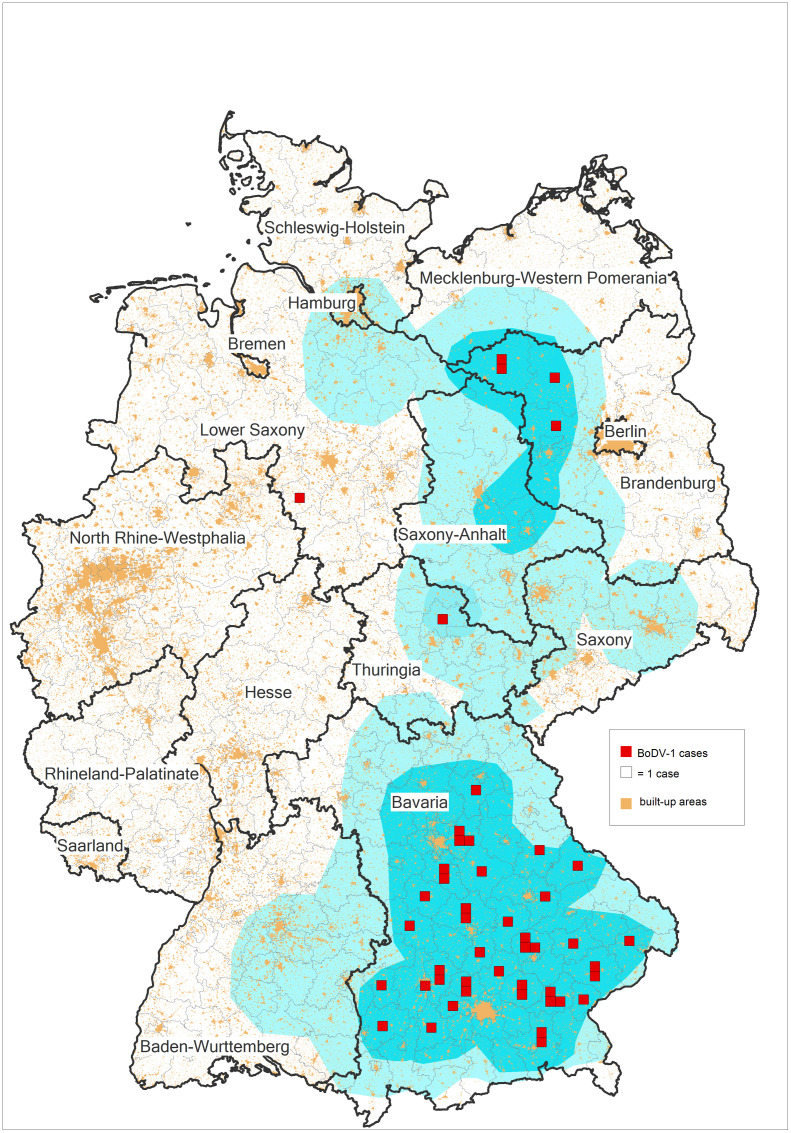
Map of places of residence of 47 human notified Borna disease virus 1 (BoDV-1) cases out of 50 known confirmed (sporadic) cases, 1992–2024. Places of residence of human cases are depicted in the center of the respective administrative district (German “Landkreis”) as red squares relative to the known endemic area of BoDV-1 (light blue) [6]. Built-up areas are depicted in orange. The figure was created with GfK RegioGraph.

In 2018, BoDV-1 was demonstrated to cause severe and mostly fatal encephalitis [1,2], following a long and controversial scientific debate on BoDV-1 pathogenicity in humans [3]. In veterinary medicine, BoDV-1 encephalitis had long been known as Borna disease, affecting mainly horses and sheep [3,4]. Mandatory reporting of direct pathogen detection in human cases was introduced in 2020 in Germany, and active case finding [5], and an increased awareness among clinicians led to the identification of 50 molecularly confirmed (partially retrospective) sporadic human cases as of December 2024 (source: Robert Koch Institute), with a focus on Bavaria (Fig 1). A few more confirmed cases (among them at least one retrospective case) have been detected in the current year 2025. Almost all cases (49/50) were fatal. However, ever since the first description of human BoDV-1 encephalitis, epidemiological and medical studies on this severe zoonotic disease have been challenging. This is mainly due to the low incidence with an estimated maximum of 10 incident cases per year (5 confirmed incident cases in 2022, 5 in 2023, 1 in 2024, Source: Robert Koch Institute as of December 31, 2024), case restriction to parts of Germany [6], assumed low level of awareness, and the variable, but often short disease course of 32 (IQR 21-41) days in median after initial hospitalization [7].

Thus, powerful prospective epidemiological, clinical, or therapeutic studies are virtually unfeasible. In recent years, though, larger (retrospective) case series and smaller studies on the epidemiology and phylogeny [4,8], the clinical and paraclinical picture and diagnostics [5,7,9–11] as well as therapeutic attempts [7] helped to increase our knowledge.

## The current epidemiological characteristics limit possible preventive measures

BoDV-1 infections of dead-end hosts such as humans (and other non-reservoir hosts like horses) are the result of spill-over transmission from a reservoir host, the bicolored white-toothed shrew (*Crocidura leucodon*) shedding the virus in various excretions [3]. BoDV-1-endemic regions have been identified in Germany, Switzerland, Austria, and Liechtenstein, [3,4,12]. However, transmission events or transmission routes remain unknown or at least uncertain: A large case-control study could not identify any potential transmission event or risk factor other than a rural place of residence on the fringe of a settlement close to nature with all cases living in cities or communities with less than 41.000 inhabitants [8]. Consequently, it is challenging to propose any preventive measures. Transmission likely occurs in the peridomestic area, covertly for the infected person, and possibly indirectly from the environment contaminated with shrew excretions containing the virus [4,8].

## Key characteristics of BoDV-1 pathogenesis—Persistent infection, immune-mediated disease, and the lack of an effective therapy

Several studies indicate that inapparent or mild clinical courses of BoDV-1 infection are unlikely; the disease rather presents as fulminant encephalitis with almost always fatal outcomes [7,13,14]. Experimental animal models have shown that BoDV-1 can enter the host via the nasal mucosa or by subcutaneous injection, followed by retrograde intraaxonal transport (for example, along the olfactory route) to the central nervous system (CNS) [15,16].

BoDV-1 establishes persistent, non-cytopathic infection (reviewed in [17,18]), but triggers an inflammatory reaction with mononuclear infiltration of the brain, paralleled by astrocyte and microglia activation with the release of pro-inflammatory cytokines, and a widespread destruction of human brain tissue [19,20]. Consistently, animal models have demonstrated the encephalitis to result from an immunopathogenesis mediated by virus-specific T-lymphocytes [21,22].

Unawareness among clinicians, a facultative and unspecific prodromal phase in which lumbar puncture or imaging is typically not carried out, late seroconversion and the progression to coma within 3 days in median (IQR 2-5) after hospitalization hinders diagnosis early in the disease course [7,10] or even after potential exposure, such as shrew bites, or contact with shrew excretions. Sensitive tests, such as RT-PCR from CSF, demand awareness in the first place and are often ordered far too late. Thus, likely due to late diagnosis and the markedly progressed disease state, sustainable clinical improvement under experimental therapy could not be seen, and treatment remained restricted to individual attempts [7]. Treatment recommendations regarding substance and dosage for BoDV-1 encephalitis therapy (or even post-exposure prophylaxis) in humans are lacking.

## Could the strategy in rabies prevention be a role model for BoDV-1?

For infectious diseases with limited preventive measures, lack of therapy, and high fatality, the prevention by vaccination is an option to wish for. Human rabies, caused by rabies virus, is another zoonotic infectious disease with striking similarities to human bornavirus encephalitis, both viruses belonging to the same order (*Mononegavirales*), resulting in nearly universal fatality rates after traveling to the CNS and causing severe encephalitis, and both having no curative treatment option.

There are, however, also several important differences regarding prevention: (i) Rabies is widespread in many parts of the world with a large population at risk, whereas BoDV-1 exhibits a small target group for vaccination [4]. (ii) For rabies, potential exposure events (i.e., dog bites in endemic regions) are defined and mostly clearly identifiable, but remain completely unclear for BoDV-1 [8]. Formulating indications for post- (or even pre-) exposure prophylaxis, therefore, seems impossible for BoDV-1, whereas the specific nature of potential exposure to rabies determines the type of post-exposure prophylaxis as well as the indication for pre-exposure vaccination. (iii) A very effective and safe vaccine is available to prevent rabies in humans. Furthermore, vaccinating the reservoir foxes effectively eradicated terrestrial rabies in parts of the world, while eradicating BoDV-1 in shrews—a protected species—would require highly unconventional strategies and would be a complex endeavor. Although the comparison between BoDV-1 and rabies seems obvious, it is misleading when it comes to administering a protective vaccine.

## A future safe and efficacious BoDV-1 vaccine—Likely a challenging endeavor

The idea of developing a (animal) BoDV-1 vaccine already existed in Germany among veterinarians in the early 20^th^ century after repeated outbreaks in horse and sheep husbandries [23]. A supposedly attenuated live vaccine passaged in rabbit brains was used to vaccinate animals in the endemic regions of mainly East Germany over years. However, potentially insufficient attenuation, questionable efficacy, concerns regarding possible post-vaccination shedding at that time, and the collapse of East Germany led to the suspension of the animal vaccination in 1992 [24].

Several experimental approaches to develop a vaccine against bornaviruses in animals yielded variable results regarding efficacy against persistent infection and (fatal) disease as well as vaccine-triggered exacerbation of the disease and were not pursued any further. Protective immunity was achieved in immunocompetent mice, but not in animals deficient for CD8-positive T-lymphocytes [25]. This is in line with previous studies demonstrating T-lymphocytes to mediate protection against BoDV-1 infection, while humoral immunity appears to play a minor role (reviewed in [21,22]). Given the potential of persistent BoDV-1 infection and the known immunopathogenesis, a prophylactic vaccine must provide a robust (likely T-cell-driven, mucosal) immunity to reliably eliminate the virus immediately at the site of entry or at least before it enters the CNS. In contrast, an immune response providing only incomplete protection against the infection might even result in enhanced immunopathology [22]. It remains to date questionable, if sterile immunity preventing both, the infection and the disease, can be achieved with sufficient certainty. T-cells seem to be responsible for both, protection against the infection as well as immunopathogenesis of the disease (reviewed in [22]).

## The target population—Whom to vaccinate?

In addition to these challenges in vaccine development, there are challenges regarding the target population: Post-exposure vaccination of persons known to be exposed is non-practical for BoDV-1, as the exposure event usually remains elusive. For pre-exposure vaccination, taking present census and level of urbanization data into account, as well as the currently known endemic area of BoDV-1, an estimated 5–8 million people in rural localities of parts of Germany, Austria, Switzerland, and Liechtenstein could theoretically be at risk for a BoDV-1 infection. However, given the rarity of BoDV-1 encephalitis, likely only a minor fraction of this population might actually be exposed to the virus on the basis of known or unknown risk or host factors. Real-world effectiveness data (as obtained for SARS-CoV-2 vaccinations) with massive infections will not be obtainable for BoDV-1 for obvious reasons. The entire population living rurally in virus-endemic areas would have to be vaccinated to prevent a small absolute number of infections. The number needed to vaccinate, a widely used number to quantify immunization benefits, would be very large to reduce one human case only. This emphasizes the need for the development of a vaccine with an extremely high safety profile and extensive preclinical and clinical testing, raising also economical and ethical questions.

## Conclusion

Both, humans and animals would certainly benefit from a vaccine against BoDV-1, especially as long as there is no causal therapy and the infection is almost universally fatal. However, the complex immunological mechanisms of BoDV-1 complicate vaccine development. Even if there was a safe and efficacious vaccine, targeted application of either pre- or post-exposure vaccination would seem unrealistic since exposure events remain unknown. Thus, at this point in time, awareness campaigns, the development of novel antivirals and concerted and standardized (theory-based) therapy recommendations although also limited remain the current focus to improve human BoDV-1 control.
